# c-di-GMP Enhances Protective Innate Immunity in a Murine Model of Pertussis

**DOI:** 10.1371/journal.pone.0109778

**Published:** 2014-10-15

**Authors:** Shokrollah Elahi, Jill Van Kessel, Tedele G. Kiros, Stacy Strom, Yoshihiro Hayakawa, Mamoru Hyodo, Lorne A. Babiuk, Volker Gerdts

**Affiliations:** 1 Faculty of Medicine and Dentistry, University of Alberta, Edmonton, Alberta, Canada; 2 Vaccine and Infectious Disease Organization, International Vaccine Centre, University of Saskatchewan, Saskatoon, Saskatchewan, Canada; 3 Department of Veterinary Microbiology, Western College of Veterinary Medicine, University of Saskatchewan, Saskatoon, Saskatchewan, Canada; 4 Faculty of Engineering, Department of Applied Chemistry, Aichi Institute of Technology, Toyota, Japan; Instituto Butantan, Brazil

## Abstract

Innate immunity represents the first line of defense against invading pathogens in the respiratory tract. Innate immune cells such as monocytes, macrophages, dendritic cells, NK cells, and granulocytes contain specific pathogen-recognition molecules which induce the production of cytokines and subsequently activate the adaptive immune response. c-di-GMP is a ubiquitous second messenger that stimulates innate immunity and regulates biofilm formation, motility and virulence in a diverse range of bacterial species with potent immunomodulatory properties. In the present study, c-di-GMP was used to enhance the innate immune response against pertussis, a respiratory infection mainly caused by *Bordetella pertussis*. Intranasal treatment with c-di-GMP resulted in the induction of robust innate immune responses to infection with *B. pertussis* characterized by enhanced recruitment of neutrophils, macrophages, natural killer cells and dendritic cells. The immune responses were associated with an earlier and more vigorous expression of Th1-type cytokines, as well as an increase in the induction of nitric oxide in the lungs of treated animals, resulting in significant reduction of bacterial numbers in the lungs of infected mice. These results demonstrate that c-di-GMP is a potent innate immune stimulatory molecule that can be used to enhance protection against bacterial respiratory infections. In addition, our data suggest that priming of the innate immune system by c-di-GMP could further skew the immune response towards a Th1 type phenotype during subsequent infection. Thus, our data suggest that c-di-GMP might be useful as an adjuvant for the next generation of acellular pertussis vaccine to mount a more protective Th1 phenotype immune response, and also in other systems where a Th1 type immune response is required.

## Introduction

Pertussis is one of the most serious childhood diseases responsible for hundreds of thousands of deaths every year. The disease is primarily caused by infection with the Gram-negative bacterium *Bordetella pertussis and* occasionally by *Bordetella parapertussis*. More recently, cases of *Bordetella holmesii* have been identified during pertussis outbreaks that have mainly affected adolescents [1]. Pertussis is characterized by severe respiratory symptoms including paroxysmal cough and apnea. The infection is mediated via inhalation of aerosol droplets and typically remains localized in the respiratory tract [2]. However, in immunosuppressed subjects the disease can progress to generalized bacteremia [3].

Over the past two decades, a resurgence of pertussis in infants and a shift of cases from children to adolescents/adults have been reported worldwide [4], [5]. The immune response against *B. pertussis* relies on both specific and innate immune responses. Although specific correlates of protection have not yet been fully established, the use of vaccines over decades has proven to be highly effective in reducing the disease. Both acelluar pertussis vaccines (Pa) and the whole-cell pertussis vaccines (Pw) are currently available and various studies have demonstrated the importance of vaccine-induced antibodies against *B. pertussis* virulence factors including pertussis toxin (Ptx), fimbria (fim 2 and fim 3) and pertactin [6]–[10]. However, it has been difficult to establish a direct correlation between antibody titers and protection from disease [11], [12]. Cell-mediated immunity, and in particular CD4^+^ T cells are involved in protection, especially when using Pw vaccines [13], [14]. Other reports have shown that the secretion of IFN-γ is critical for effective control of bacterial spread in the lung. For example, IFN-γ^−/−^ or IFN-γ defective mice developed disseminating lethal infections following challenge [2], [15]. Furthermore, protection may also be associated with the induction of IL-12 [16] and TNF-α required for effective phagocytosis of the bacteria [17], [18]. More recently, Th-17 cells and the secretion of IL-17 have also been implicated in cell-mediated protection from *B. pertussis* [19].

The innate immune response against pertussis involves various immune cells including macrophages, neutrophils, dendritic cells, γδ-T cells, NK cells and NKT cells [20]. The two main phagocytic cell populations that constitute pulmonary innate immunity are resident alveolar macrophages and recruited neutrophils [21]. In addition, local and rapidly recruited lung dendritic cells have been demonstrated to internalize bacteria and promote the expression of type 1 cytokines by NK cells, T cells, and NKT cells [22]–[26], which then directly impact specific immune responses. Here, we evaluated the potential of cyclic diguanylate (c-di-GMP; 3′, 5′-cyclic diguanylate) to act as innate immune stimulator.

C-di-GMP was first identified in Gluconacetobacter xylinum and Agrobacterium tumefaciens. The molecule is involved in various intracellular signaling pathways including cellulose biosynthesis [27]–[29], bacterial growth, cellular adhesion, cell-surface interactions and biofilm formation [30]–[34]. c-di-GMP is found at concentrations of 5–10 µM in many bacterial species, but not in eukaryotes [35]–[37]. Recently, a number of studies have shown that the innate immune response elicited by c-di-GMP is a potent immunomodulator for the treatment of bacterial infections and can act as an immune modulator and immunostimulatory molecule [32], [38]. For example, intramammary administration of c-di-GMP has been shown to prevent colonization and biofilm formation by *Staphylococcus aureus* in a murine model of mastitis [39]. In another study, intranasal or subcutaneous administration of c-di-GMP stimulated a robust innate immune response characterized by increased recruitment of immune cells into the lung which resulted in protection against intratracheal challenge with *Klebsiella pneumonia* in mice [32]. Furthermore, intranasal administration of c-di-GMP significantly reduced nasopharyngeal *Streptococcus pneumoniae* colonization by induction of a robust but transient proinflammatory response characterized by production of cytokines, chemokines and recruitment of pulmonary DCs [40]. Intraperitoneal administration of c-di-GMP in mice enhanced monocyte and granulocyte recruitment [38]. Interestingly, stimulation of human immature dendritic cells with c-di-GMP resulted in an increase in the expression of costimulatory molecules CD80/CD86 and maturation marker CD83, MHC class ΙΙ, cytokines, chemokines and chemokine receptors [38]. More recently, STING has been identified as a direct detector of cyclic dinucleotides, provides more insight into the fundamental mechanisms by which the innate immune system can detect bacterial infection [41]. Here, we demonstrate that intranasal administration of c-di-GMP in mice resulted in induction of protective immune responses against bacterial challenge with *B. pertussis*. These results also confirm previous observations that innate immunity plays an important role in disease protection against *B. pertussis* infection.

## Materials and Methods

### Mice

Female specific pathogen free BALB/c mice were obtained at 5–7 weeks of age from the Charles River Institute. All animals were maintained under specific pathogen free conditions within the animal care unit of VIDO until the day of sacrifice. This study was carried out in strict accordance with the recommendations in the Guide for the Care and Use of Laboratory Animals of the Canadian Council for Animal Care. The protocol was approved by the Committee on the Ethics of Animal Experiments of the University of Saskatchewan. All surgery was performed under sodium pentobarbital anesthesia, and all efforts were made to minimize suffering.

### c-di-GMP

The c-di-GMP used in our study was chemically synthesized and prepared as described previously [42]. The purity of the c-di-GMP used in this study was>98% and was confirmed by HPLC, ^31^P-NMR, and ESI-TOF Mass Spectophotometrical analysis. The peptides used in this study were free of endotoxin contamination as determined by the Limulus assay. Control c-GMP (Sigma) or c-di-GMP was reconstituted at a concentration of 200 µM in 30 µl of sterile saline for treatment of mice.

### Bacterial culture

Bacterial suspensions of strain Tohama Ι were stored at −70°C in Casamino acid plus 10% glycerol. Organisms were grown on the surface of Charcoal agar (Becton Dickinson and Company, USA) containing 10% (vol/vol) defibrinated sheep blood and 40 µg/ml of Cephalexin (Sigma-Aldrich, USA) at 37°C for 48 hr. Bacteria were harvested from plates by scraping off and resuspending bacteria in Stainer-Scholte (SS) medium. Bacteria were washed by centrifugation at 2500 g for 10 min. The pellets were resuspended in phosphate buffered saline (PBS; pH 7.2) and adjusted to the indicated optical density (OD) at 600 nm using a spectrophotometer (Ultrospec 3000, Pharmacia Biotech, U.K). The bacterial suspension was kept on ice until it was used for the challenge. The corresponding viable counts of these suspensions were determined by plating serial dilutions of the bacterial suspension onto Charcoal agar plates and incubation at 37°C for 4–5 days.

### Respiratory challenge

Mice were lightly anaesthetized while a single drop of 30 µl c-di-GMP, the nucleotide control c-GMP or PBS (0.1 M, pH 7,2) was administered intranasally (i.n.) 24 hr prior to bacterial challenge. For challenge mice were anaesthetized and an inoculum containing 1–3×10^7^ *B. pertussis* was carefully placed on the top of each nostril, and allowed to be inhaled [43]. After the challenge at days 2, 4 and 6, lungs were removed from mice and placed in SS medium or PBS (0.1 M, pH 7,2) containing 0.4 mM of irreversible serine protease inhibitor APMSF (4-Amidinophenyl-methanesulfonyl fluoride; Roche Diagnostics GmbH, Germany).

### Whole lung homogenization for bacterial and cytokine determination

The whole lung from each mouse was homogenized in 2 ml of SS medium. Bacterial counts were assessed by plating serial dilutions of the homogenate onto Charcoal agar plates and incubation at 37°C for 4–5 days. For assessment of cytokine levels the whole lungs were homogenized in 2 ml of PBS containing APMSF for cytokine determination and flow cytometric analysis. The lung homogenates in PBS with protease inhibitor were then centrifuged at 1200xg for 10 min. Supernatants were collected and stored at −20°C for measurement of cytokine levels.

### Detection of cytokine levels by ELISA

Concentrations of murine IFN-γ, TNF-α, IL-4, IL-6, IL-12, IL-17, IL-23 and MCP-1 were measured in serum and lung homogenate supernatants using an Endogen, Opteia or capture Duoset ELISA development system. Immunolon 2-HB 96-well plates were coated with anti-mouse IFN-γ (4 µg/ml), anti-mouse TNF-α (0.8 µg/ml), anti-mouse IL-4 (4 µg/ml), anti-mouse IL-6 (2 µg/ml), anti-mouse IL-12p70 (4 µg/ml), anti-mouse IL-23 (2 µg/ml; R & D Systems), anti-mouse IL-17 (2 µg/ml; eBioscience) and anti-mouse MCP-1 (1∶250; BD Biosciences) overnight at 4°C. Prior to use, the plates were blocked with PBS and 1% bovine serum albumin (BSA) for 1 h at room temperature (RT). Samples were added to the wells in a volume of 50 µl plus 50 µl of PBS-1% BSA and incubated for 2 h at RT. The reaction was amplified with biotinylated monoclonal antibodies to murine IFN- γ (0.4 µg/ml), TNF-α (0.2 µg/ml), IL-4 (0.4 µg/ml), IL-6 (0.2 µg/ml), IL-8 (25 µg/ml), IL-10 (50 µg/ml), IL-12p70 (0.4 µg/ml), IL-23 (Quantikine kit; all from R & D Systems), IL-17 (0.5 g/ml; eBioscience) and MCP-1 (1∶250; BD Biosciences). Plates were incubated for 1 h at RT. Detection was carried out with peroxidase-conjugated streptavidin (1∶5000; Jackson Laboratories) following 60 min incubation at RT and reaction was visualized with p-nitrophenylphospate (Sigma-Aldrich). Standard curves were generated using recombinant murine IFN- γ, TNF-α, IL-4, IL-6, IL-12, IL-17, IL-23 and MCP-1.

### Quantification of nitric oxide (NO) in the lung

Lung homogenates were collected and centrifuged down at 10000 g for 20 min before the assay. The production of NO was determined by measuring the accumulation of nitrite, the stable metabolite of NO, in homogenate supernatants using the Griess reaction (Colorimetric assay kit, Alexis Biomedicals, Switzerland) as previously described [44]. A standard nitrite curve was generated in the same fashion using NaNO_2_.

### Lung leukocyte isolation

To remove intraepithelial lymphocytes (IEL) lung tissues were treated with 20 ml PBSA-buffer (0.1 M, pH 7,2) with 2 mM EDTA, pH 7.3 for 45 min on stir at 37°C. To remove lamina propria lymphocytes (LPL), lung tissues were enzymathically digested in digestion buffer (20 mM PBS containing 5% fetal bovine serum, 1% Hepes, 1% antibiotic/antimycotic and 250 U/ml collagenase type V) for 45 min on stir at 37°C. The total lung cell (IEL and LPL) suspension was, pooled, pelleted, resuspended, and centrifuged through a 30%, 40% and 60% percoll gradient to enrich for leukocytes for flow analysis. Cell counts and viability were determined on a hemocytometer using trypan blue exclusion.

### Messenger RNA expression analysis using real-time PCR

The whole lung was removed at different time points and immediately snap frozen in liquid nitrogen and then stored at 70°C for further RNA extraction. Total RNA was extracted from 30 to 50 mg of tissue using the RNeasy Mini kit (Qiagen, Mississauga, Canada). Total RNA quantity and purity were determined by optical density (OD) at 260 and 280 nm wavelengths using a spectrophotometer (Ultrospec 2000, Pharmacia Biotech, Baie d'Urfe, PQ). RNA samples were treated with DNAse I Amp Grade (Invitrogen) (1 U/µg of RNA). The absence of genomic DNA contamination was validated by use of treated RNA as template directly in PCR. One microgram of total RNA was used for a reverse transcription reaction (total 21 µl) with Oligo(dt)_12–18_ primers and SuperScript II reverse transcriptase (SuperScript first strand synthesis system for RT-PCR (Invitrogen) for evaluation of relative expression. Negative controls were made by replacing the reverse transcriptase with diethyl pyrocarbonate-treated water. The resulting single-stranded cDNA was then used in for real-time PCR (qPCR) analysis (iCycler iQ Real-Time PCR detection system, Bio-Rad, Hercules, CA) for evaluation of relative expression. cDNA was combined with primer/probe sets and IQ SYBR Green Supermix (Bio Rad) according to the manufacturer's recommendations. Primers for mouse beta defensin 3 (mBD3) forward (5′-TCTGTTTGCATTTCTCCTGGTG-3′), reverse (5′-TAAACTTCCAACAGCTGG-AGTGG-3′) and mBD4 forward (5′-TCTGTTTGCATTTCTCCTGGTG-3′), reverse (5′-TTTGCTAAAAGCTGCAGGTGG-3′) and mouse macrophage-inflammatory protein (mMIP-2), forward, 5′-GAA CAT CCA GAG CTT GAG TGT GA-3′, reverse, 5′-CCT TGA GAG TGG CTA TGA CTT CTG T-3′, respectively. The PCR conditions were 95°C for 3 min, followed by 45 cycles with denaturation at 95°C for 15 s, annealing temperature for 30 s, and elongation at 72°C for 30 s. The specificity of the PCR reactions was assessed by the analysis of the melting curves of the products, size verification and sequencing of the amplicons. Samples were normalized by using the average cycle threshold (CT) of glyceraldehyde-3-phosphate dehydrogenase (GAPDH) as a reference in each tissue. The suitability of GAPDH as a reference was confirmed by the lack of variation observed between animals from a same group. Relative quantitation of mRNA levels was plotted as fold-increase compared to untreated control lungs.

### Flow cytometric analysis

Using FITC or PE-labeled antibodies (BD Pharmingen, San Diego, CA), isolated cells were stained with anti-CD4, anti-CD8, anti-αß-Tcr, anti-γδ-Tcr, anti-DX5 (NK cell marker), anti-CD11c, CD40, CD83, CD86 and their isotype controls. Cells were collected on a FACS Calibur cytometer (BD) using Cell Quest software (BD).

### *In vitro* growth inhibition assays

The sensitivity of *B. pertussis* to c-di-GMP was compared with synthetically derived porcine cathelicidin protegrin 1 (PG1) and human cathelicidin LL-37 by co-culturing appropriate concentrations of c-di-GMP, PG1 and LL-37 in 20 mM PBSA (280 µl) with 5–7×10^6^ CFU (10 µl) bacteria as previously described [45]. Plates were incubated at 37°C for 2 hrs. Supernatants were plated onto Charcoal agar plates to evaluate the number of viable bacteria.

### Statistical analysis

All outcome data from this study followed non-normal distributions. To account for this, outcome data were ranked and then an ANOVA or a Student's t-test was used to detect differences amongst the experimental groups. The distributions of the ranked data and the residuals from each ANOVA were consistent with the assumptions of procedure. If there were more than two experimental groups in the analysis and the ANOVA was significant, the means of the ranks were compared using Tukey's test. Probabilities less than or equal to 0.05 were considered significant.

## Results

### c-di-GMP pretreatment reduces bacterial load in lungs of infected mice

**Figure 1:**
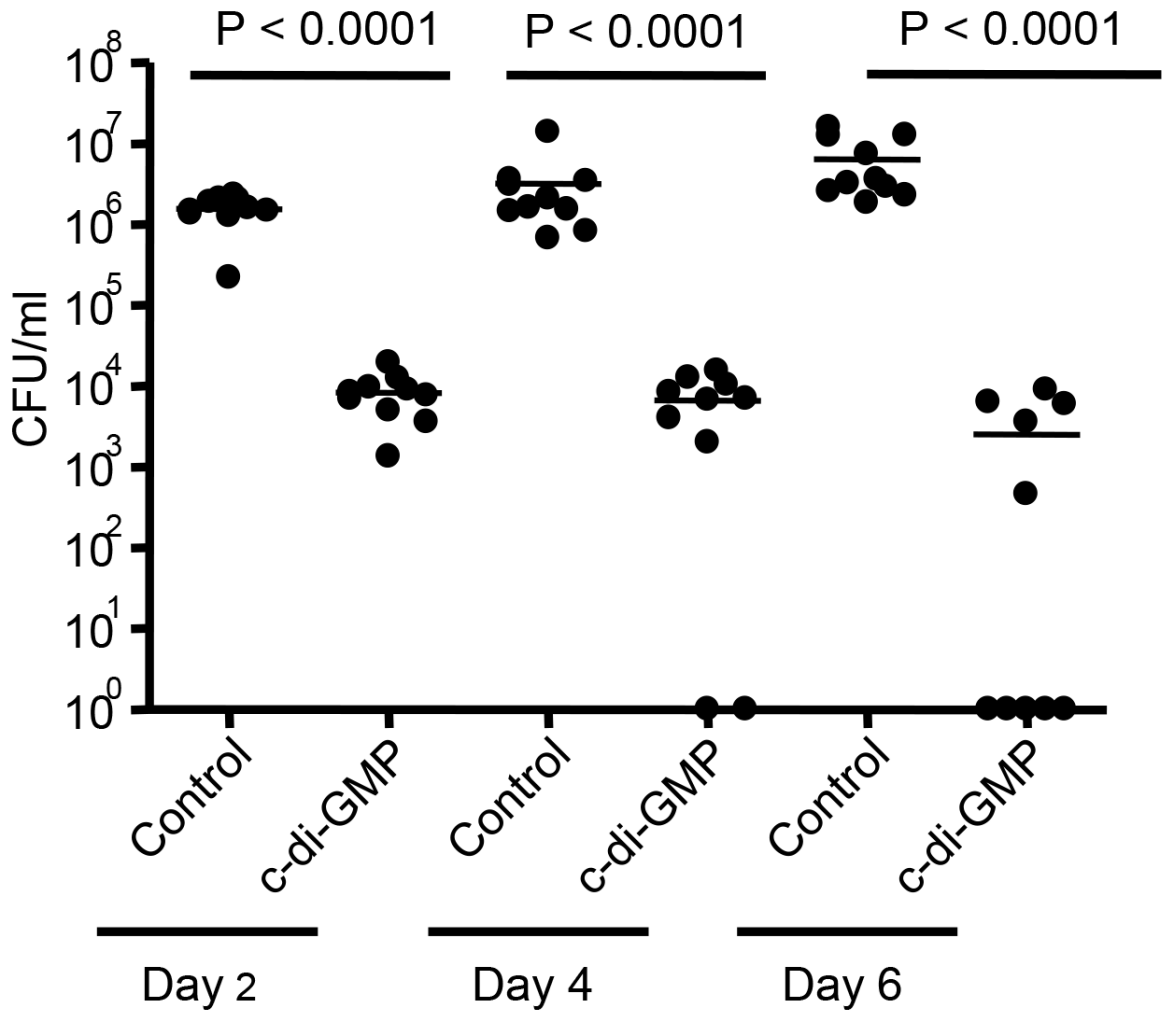
Pretreatment with c-di-GMP reduces the bacterial load in the lungs of mice challenged with *B. pertussis*. Mice were intranasally treated with c-di-GMP or control (PBS) at 24 hr prior to challenge. Bacterial counts were determined at day 2, 4 and 6 post challenge. The results are expressed as the mean values ± the standard deviations per lung, as counted from individual lungs of 10 mice per group from three separate experiments. *, P<0.0001 in lung homogenates versus the control group.

Balb/c mice were either treated intranasally with c-di-GMP (200 nM) or PBS and 24 hr later intranasally challenged with 1–3×10^7^ CFU *B. pertussis*, strain Tohama I. Bacterial counts were determined at days 2, 4 and 6 post challenge. As shown in Fig. 1, intranasal treatment with c-di-GMP resulted in a significant reduction of bacterial counts in lungs of infected mice at 2, 4 and 6 days post challenge (p<0.0001). In some experiments C-GMP instead of PBS used as control but no effects on *B. pertussis* colonization was noted (data not shown).

### c-di-GMP treatment increases cell recruitment to the lung

**Figure 2:**
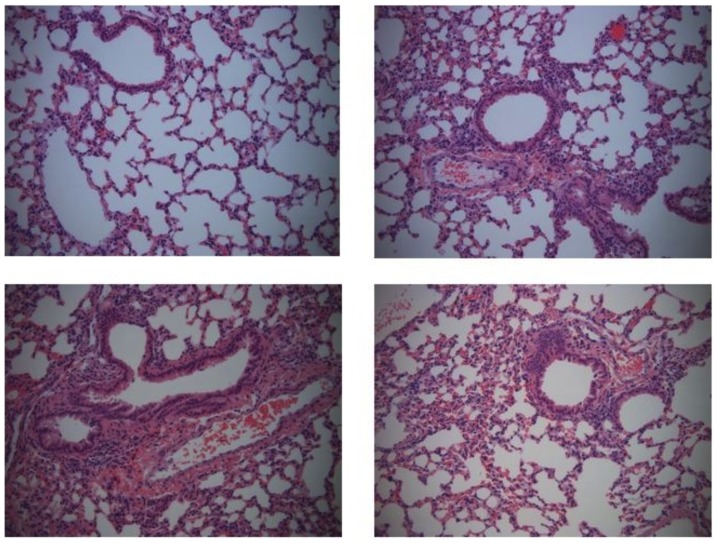
Infiltration of neutrophils and macrophages in c-di-GMP treated animals. (A) Photomicrograph of lung biopsies taken from a control mouse at 24 hr post treatment with c-GMP, obtained with a 10 X objective plus digital zoom. (B-D) Photomicrographs of lung biopsies taken from three different mice at 24 h post treatment with c-di-GMP (10 X objective plus digital zoom).

To address whether treatment with c-di-GMP enhanced recruitment of immune cells to the lung, mice were intranasally treated with either c-di-GMP or equal concentrations of nucleotide control c-GMP. 24 hr later their lungs were removed and cell recruitment into the lung was assessed by histology using H& E staining. As shown in Figure 2, administration of c-di-GMP resulted in increased numbers of neutrophils and macrophages recruited into alveolar walls and spaces, as well as perivascular lymphoid infiltrations. Flow cytometry at 1, 2, 4 and 6 days post challenge revealed that c-di-GMP administration resulted in a fivefold increase of CD11c+ cells and about a fivefold increase of NK cells expressing DX5 compared to control mice (Table 1). No differences in the number of total αß-T cells or γδ-T cells were observed between the two groups (data not shown).

**Table 1:** The effects of c-di-GMP on cellular recruitment and activation in the lung following c-di-GMP treatment.

Cell population	c-di-GMP-treated	Control
DX5+ (NK)	29.1%	6.2%
CD11c	10.2%	2.0%
γδ TCR	6.0%	2.0%
CD86	54.4%	33.0%

### Enhanced cytokine and chemokine production following treatment with c-di-GMP

**Figure 3:**
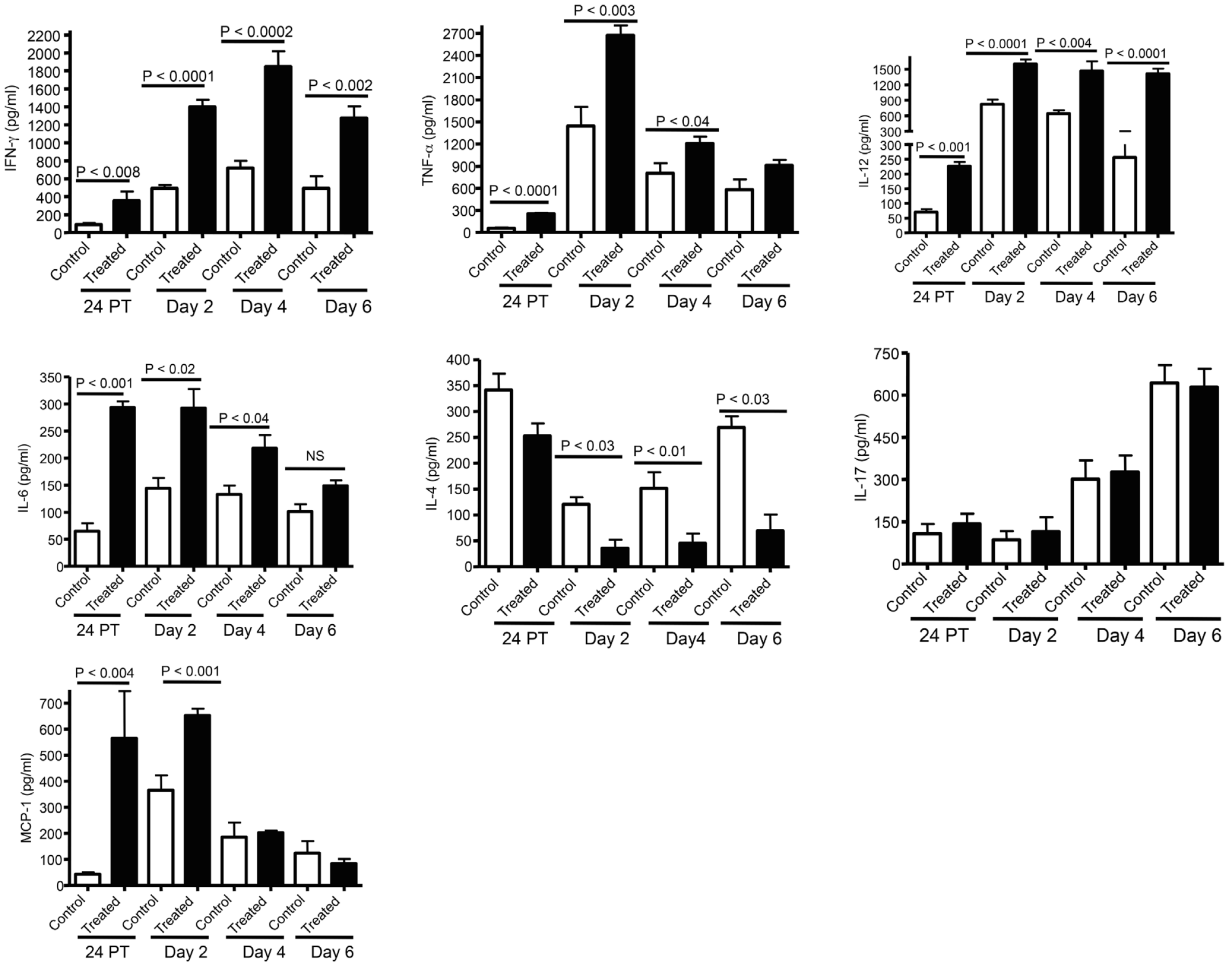
Cytokine (IFN-γ, TNF-α, IL-12p70, IL-6, IL-4 and IL-17) and chemokine (MCP-1) concentrations in whole lung homogenates following c-di-GMP treatment and intranasal challenge infection with *B. pertussis*. Mice were treated with c-di-GMP (black bars) or control c-GMP (white bars) at 24 hr prior to challenge infection. Whole lungs were removed and cytokine concentrations determined in lung homogenate supernatants at 24 hr post treatment (24PT) and days two, four and six post challenge (Day 2–6). The results shown are as the means ± SD of cytokine and chemokine concentrations detected by ELISA from 3 separate experiments and nine to twelve animals per group.

**Figure 4:**
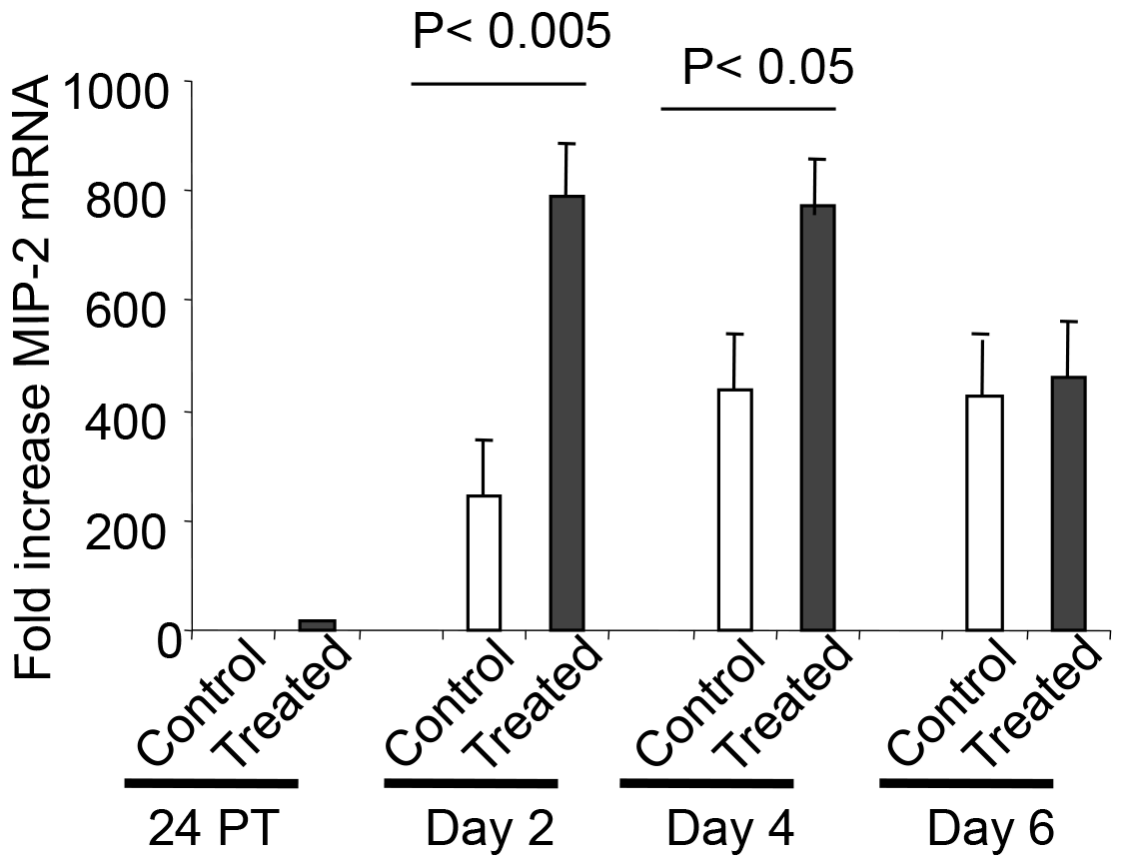
MIP-2 mRNA levels in lung tissues following c-di-GMP treatment. Mice were treated intranasally with c-di-GMP or control c-GMP at 24 hr prior to challenge infection. MIP-2 mRNA levels in lung were determined prior to challenge and at days 2, 4 and 6 post challenge by real-time PCR. Values shown represent the fold increase of the mean compared to non-infected control mice (five to eight animals per group).

To define the potential mechanisms of enhanced dendritic and NK cell recruitment, we assessed the induction of cytokines and chemokines following treatment with c-di-GMP and challenge infection with *B. pertussis*. Administration of c-di-GMP resulted in significant production of IFN-γ, TNF-α, IL-12p70 and IL-6 in the lungs of mice 24 hr post treatment (p≤0.008) and at days 2, 4 and 6 post challenge (P≤0.004; Fig. 3). In contrast, pretreatment with c-di-GMP reduced IL-4 production (P≤0.03) at days 2, 4 and 6 post infection, but did not significantly alter the expression of either IL-17 or IL-23 (Fig. 3 and data not shown). However, pre-treatment with c-di-GMP significantly enhanced the production of MCP-1 prior to and at 24 hr post infection (P<0.004; Fig. 3) as well as the expression of MIP-2, mRNA levels (>3 fold increase; Fig. 4) at days 2 and 4 post challenge (P<0.005 and P<0.05 respectively). No changes in the expression of beta defensin 3 (BD-3) and beta defensin 4 (BD-4) were observed.

### c-di-GMP treatment enhances the production of nitric oxide in vivo

**Figure 5:**
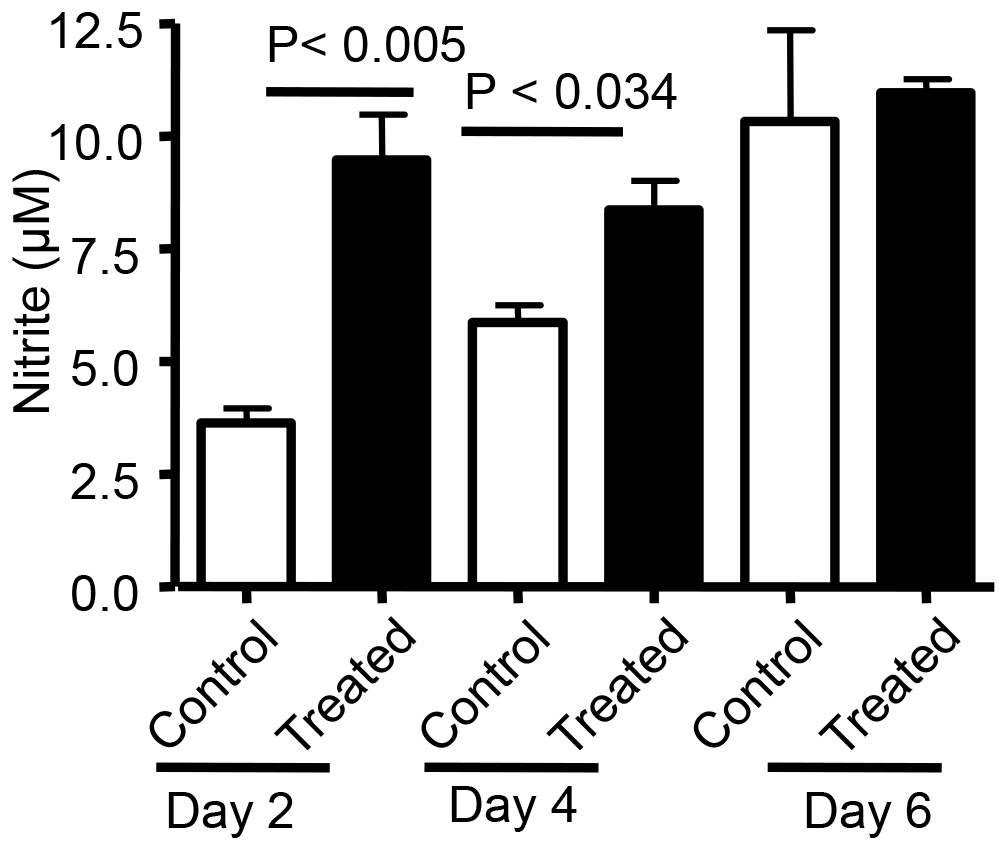
Nitric oxide production following c-di-GMP treatment. Nitrite production, as a measure of NO expression, was assessed at 24 hr after treatment with c-di-GMP and 24 hr after infection with *B. pertussis* in lungs of mice. Control animals were treated with PBS. Values shown represent the means ± SD from two independent experiments (10 animals per group).

Nitric oxide has been shown to be a necessary component of effective innate immunity against bacterial and fungal agents [44], [46]. To investigate whether the enhanced clearance of bacteria observed in c-di-GMP treated mice was associated, in part, to increased production of NO, we assessed the presence of nitrite, the stable metabolite of NO, in c-di-GMP treated or control mice at 24 and 48 hr post intranasal infection with *B. pertussis*. As shown in Fig. 5, mice treated with c-di-GMP produced significantly more NO in their lungs at 24 hr post treatment (P<0.005) and at 24 hr post challenge as compared to control animals (P<0.034). In some experiments C-GMP instead of PBS used as control but no effects on NO production observed (data not shown).

### Disease protection is not mediated by direct antimicrobial activity

**Figure 6:**
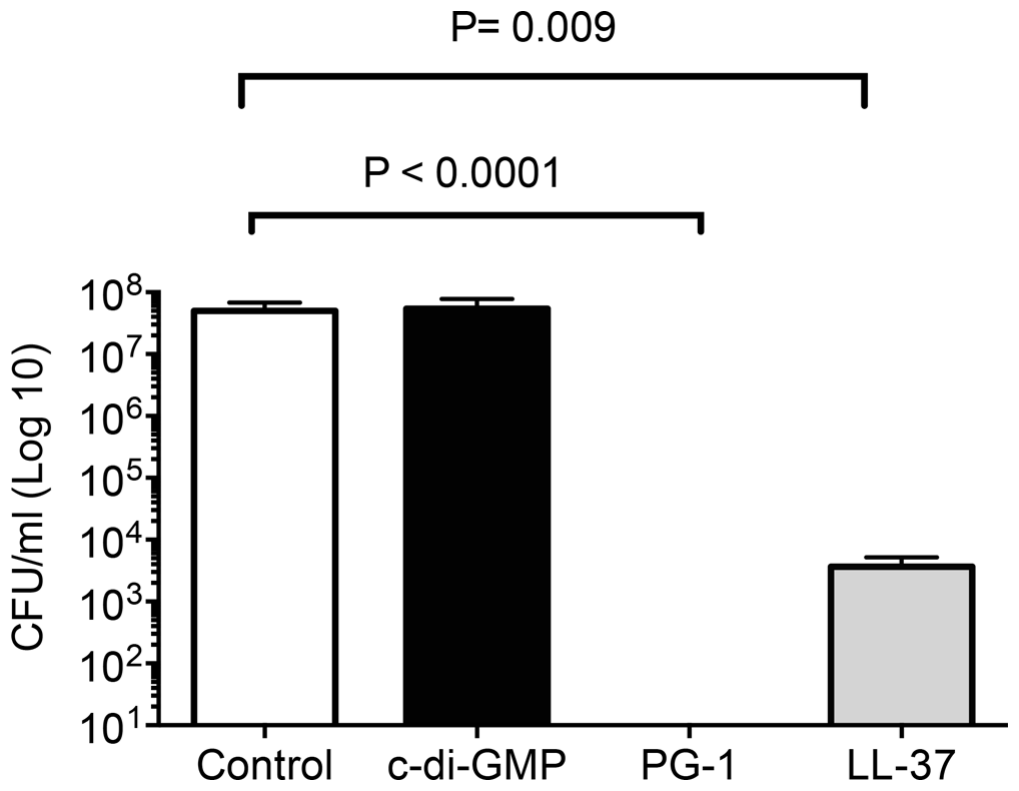
Sensitivity of *B. pertussis* to c-di-GMP in comparison to the human and porcine cathelicidins LL-37 and PG1. A total of 5×10^6^ to 7×10^6^ CFU *B. pertussis* were co-cultured in duplicates with 10 µg/ml of the respective peptide in 20 mM PBS for 2 h. Bacterial numbers were determined by plate counts. Results are form three separate experiments and expressed as the means ± SD.

The direct inhibitory effects of c-di-GMP were compared with known antimicrobial peptides (AMPs) *in vitro*. AMPs have both direct, broad-spectrum antimicrobial activity and the ability to modulate immune responses against a wide range of pathogens [47], [48]. The antimicrobial activity of c-di-GMP against *B. pertussis* was tested and compared with protegrin-1 (PG1) and LL-37, previously tested in our lab, using inhibition assays [45]. c-di-GMP was used at 5 and 150 µg/ml and PG1 and LL-37 were used at concentrations of 5–10 µg/ml. As shown in Fig. 6, *B. pertussis* was completely resistant to killing by c-di-GMP while both the porcine PG-1 and the human cathelicidin LL-37 displayed strong antimicrobial activities resulting in 1000 to 10,000,000 fold reduction of bacterial numbers (p = 0.009). Interestingly, even higher concentrations of c-di-GMP as well as prolonged incubation did not increase its inhibitory effect against *B. pertussis* (data not shown).

## Discussion

In the present study we demonstrated that intranasal treatment of mice with c-di-GMP resulted in the induction of strong respiratory immune responses against infections with *B. pertussis*. These responses were characterized by an early expression of MIP-2, a potent neutrophil recruiter, and MCP-1, a chemokine involved in the recruitment of monocytes, NK, iDCs and B cells. The immune responses were skewed towards a Th1-type response, which was further enhanced by challenge infection with *B. pertussis*. In contrast, c-di-GMP treatment reversed the induction of IL-4 production after bacterial infection. These findings are in line with previous studies indicating that Th1-helper T cells and their cytokines are necessary for effective clearance of *B. pertussis* [2], [20], [49]–[51]. Our results are consistent with recent studies in mice showing that treatment with c-di-GMP stimulates protective innate immune response and provides protection against *Klebsiella pneumoniae* and *Streptococcus pneumoniae* challenge infections [32], [40]. c-di-GMP was also proven efficacious in enhancing bacterial clearance of *S. aureus* in mice [33]. In addition, treatment with c-di-GMP resulted in enhancement of MCP-1 production, which may have contributed to increased recruitment of monocyte, macrophage, neutrophil, NK cell and DCs into the lungs of c-di-GMP treated animals. These cells have all been implicated in providing protection against bacterial pathogens including *B. pertussis* [51]. For example, NK cells are potent producers of innate IFN-γ in response to extracellular signals including the cytokines IL-12 and TNF-α [52]. NK cells provide resistance to *B. pertussis* by activating IL-12-mediated production of IFN-γ, which enhances the antibacterial activity of monocytes/macrophages, but also promotes the differentiation of Th1 cells [20]. The ability of c-di-GMP to enhance Th1 type immune response supports the direct priming of DCs [38], [40]. It has been shown that c-di-GMP activates p38 mitogen-activated protein kinase and results in enhanced IL-12 expression by DCs [32], [38]. Although, immune cells other than DCs could contribute to enhanced IL-12 production including macrophages, we propose that direct priming of DCs by c-di-GMP induces IL-12 production which stimulates IFN-γ secretion possibly from NKs. *B. pertussis* infection is more severe in IFN-γ^−/−^ mice [2], whereas *B. pertussis* disseminates from the lungs and causes organ failure in IFN-γ R^−/−^ mice [15]. Although the precise role of NK cells in this study has not been investigated in details, based upon similar studies its possible to suggest that NK cells through secretion of IFN-γ might play a significant role in bacterial dissemination, pathology and bacterial clearance in mouse lungs, possibly via activated macrophages [2], [15]. In addition, other studies have shown that NK cells confer resistance to *B. pertussis* by IL-12 mediated production of IFN-γ, suggesting a role for IFN-γ early in infection [20]. Moreover, a defective TNF-α response has been shown as a potential risk factor for infection with *B. pertussis* [18]. Taken together, these results indicate that clearance of infection is dependent on a combination of innate immune responses mediated by IFN-γ, IL-12, TNF-α and other Th1 type cytokines. Although, most recent findings demonstrate that both Th1 and Th17 cells contribute to the clearance of infection with *B. pertussis* in mice [53], in our studies c-di-GMP did not induce significant levels of IL-17 or IL-23.

Treatment with c-di-GMP also resulted in enhanced recruitment and activation of DCs and macrophages, possibly through increased release of chemoattractants such as MCP-1. Furthermore, the induction of enhanced Th 1 type cytokines in response to c-di-GMP treatment indicates that c-di-GMP can act directly on DCs which is in agreement with recent studies showing that c-di-GMP stimulated DC mediated immune responses by induction of cytokines, chemokines and increases the cell surface expression of maturation markers, leading to an overall Th1 type response [38].

The recruitment and/or activation of innate immune cells into the lungs prior to challenge infection likely contribute to improved bacterial clearance in c-di-GMP treated animals. Both macrophages and neutrophils have been shown to kill *B. pertussis in vitro*, however their mechanisms of killing, which may involve oxygen or nitrogen intermediates, are still not known [51]. It has been shown that NO is produced *in vitro* by alveolar macrophages stimulated with *B. pertussis* and by peritoneal macrophages following immunization with Pw or by alveolar macrophages following respiratory infection with *B. pertussis* [54]–[56]. IFN-γ can augment NO production by macrophages infected with *B. pertussis* [57]. Purified pertussis toxin has been shown to stimulate IFN-γ secretion through direct activation of T cells and subsequent NO production [58]. Moreover, production of NO by peritoneal macrophages from mice immunized with Pw has been shown to correlate with efficacy in the intra-cerebral (i.c.) challenge model of pertussis [59]. In addition, protection induced with Pw is compromised in NO synthase-deficient mice [60], which indicates reactive nitrogen intermediates may function in intracellular killing of *B. pertussis*. Therefore, enhanced production of nitric oxide in the treated mice with c-di-GMP, possibly by alveolar macrophages, and Th1 type cytokines such as IFN-γ, as inducer of iNOS may play a protective role in clearance of *B. pertussis*.

Introduction of the Pw in the 1950s significantly reduced the incidence of pertussis but was replaced by Pa in most developed countries [11]. Although, Pa is a safer vaccine, the resurgence of pertussis might be associated with suboptimal or waning immunity induced by Pa [61]. Analysis of immune responses in children demonstrated that Pa promote a Th2 type, whereas Pw preferentially induce a Th1 type immune response [62], [63]. Pa vaccines are delivered to children using alum as the adjuvant which preferentially promotes a Th2 type immune response. In the current study we have shown that c-di-GMP pretreatment increases IFN-γ expression and reduces IL-4 expression following *B. pertussis* challenge. This suggest that utilizing a Th1 type inducing adjuvant such as c-di-GMP with Pa instead of alum-adjuvant could provide more effective and protective immune response against pertussis. Therefore, it would be very advantageous to determine whether c-di-GMP could be used as an adjuvant with the Pa to induce a more protective Th1 type immune response against pertussis. Because our findings demonstrate that priming the innate immune system by c-di-GMP is capable to further skew the immune response towards a Th1 type and away from a Th2 type immune response during subsequent infection with *B. pertussis*.

Taken together, in the present study, we demonstrated that chemically derived c-di-GMP, while having no direct antimicrobial activity against *B. pertussis*, acted as an innate immune stimulator resulting in the expression of various cytokines and chemokines and activation of monocytes, granulocytes and DCs. This resulted in enhanced Th1 type immune response and improved clearance of bacteria from the lung. We suggest that c-di-GMP with such a broad biological activity can be utilized for clinical purposes in human and animals as an immunomodulator, immunoenhancer or vaccine adjuvant where a Th1 type immune response is needed. Our study also highlights the importance of the innate immune response and Th1 type cytokines in induction of protection against *B. pertussis* infection.
